# What factors explain pregnant women’s feeding intentions in Bradford, England: A multi-methods, multi-ethnic study

**DOI:** 10.1186/1471-2393-14-50

**Published:** 2014-01-28

**Authors:** Baltica Cabieses, Dagmar Waiblinger, Gillian Santorelli, Rosemary RC McEachan

**Affiliations:** 1 Bradford Institute for Health Research, Bradford Teaching Hospitals NHS Foundation Trust, Bradford Royal Infirmary England, Temple Bank House Bradford Royal Infirmary, Duckworth Lane Bradford BD9 6RJ, England, UK; 2 Universidad del Desarrollo Clínica Alemana Chile, Avenida La Plaza 680 San Carlos de Apoquindo, Las Condes, Santiago, Chile

**Keywords:** Theory of planned behaviour (TPB), Feeding intentions, Ethnicity, Multi-methods design, Breastfeeding, Bottle-feeding, Mix-feeding, UK

## Abstract

**Background:**

Using a multi-methods approach we aimed to explore the relative prediction of demographic, socioeconomic and modifiable predictors from the Theory of Planned behaviour (TPB) in explaining feeding intentions amongst a multi-ethnic sample.

**Methods:**

476 women completed a questionnaire at 28 weeks gestation. They were grouped into breastfeeding (N = 258), mixed-feeding (N = 50), bottle-feeding (N = 88) intenders, or a no clear intention (N = 88). Multinomial adjusted regressions explored the influence of modifiable TPB factors, along with ethnicity and socioeconomic status in predicting group membership. Free-text responses allowed women to elaborate on reasons behind their intention.

**Results:**

TPB factors were significant predictors of feeding intention. Women with high intention to breastfeed were less likely to report high attitudes in any other feeding alternative. Bottle-feeding intenders reported poorer self-efficacy regarding breastfeeding compared to breastfeeding intenders (prevalence rate ratio, PRR = 0.10). Mixed and bottle-feeding intenders reported greater self-efficacy for mixed-feeding (PRR = 1.80, 5.50 respectively). Descriptive norms for mixed (PRR = 13.77) and bottle-feeding (PRR = 10.68) were predictive of mixed-feeding intention. Reasons for breastfeeding intentions related to health considerations, whilst bottle-feeding reasons related to convenience. Mixed-feeding intenders reported both breast and bottle-related factors.

**Conclusions:**

Understanding modifiable predictors related to feeding intentions like TPB factors can help professionals target appropriate interventions to encourage breastfeeding.

## Background

The health benefits of breastfeeding for mother and child are well established worldwide [1]. Current recommendations are that babies should receive exclusively breast milk for the first six months of their lives [2]. The promotion of breastfeeding has become an integral part of the Child Health Strategy to reduce health inequalities [3], and initiation rates and breastfeeding prevalence at 6–8 weeks postpartum are key indicators for the child’s health and wellbeing [4]. The latest Infant Feeding Report within the UK observed an increase of initiation of breastfeeding in the last decade across all socioeconomic groups, from 70% in 2000 to 81% in 2010 [5]. However, prevalence of both exclusive and any breastfeeding still declines rapidly in the first two months [6,7]. These patterns have been observed in multiple urban cities not only within the UK [8] but also worldwide [9-11].

There is growing evidence on the relevance of the different factors affecting feeding practices after birth, for example mother’s age [12], socioeconomic status (e.g. income and occupation) [13], parity [12], newborn’s prematurity [14], lack of access to antenatal care [15], and other health problems of the mother or the infant [13]. However, most of the evidence is focused on breastfeeding and bottle- feeding, and little has been built upon mixed-feeding practice (both breastfeeding and bottle-feeding) and how it differentiates from the other two feeding practices [16-20]. The most recent feeding survey in the UK found that 14% of mothers intended to mix-feed their babies from birth, compared with 61% who intended to exclusively breastfeed and 17% who intended to exclusively bottle-feed [5], although caution should be exercised when interpreting these figures as intention was assessed retrospectively. Evidence suggests that early introduction of formula (in this paper equal to bottle-feeding) by mothers who breastfeed is a risk factor for early cessation of breastfeeding [6,21,22] and therefore needs further consideration by health professionals.

As mentioned before, previous research has highlighted the influence of demographic and socioeconomic factors on infant feeding. For example, women from low-income groups show significantly lower rates of breastfeeding initiation and duration [23-26]. Little analysis has explored existing differences in intention by specific ethnic groups, even though belonging to a minority ethnic group has proven to be closely related to socioeconomic deprivation and poor health [27,28]. This study was carried out in Bradford in the North of England, a city in which the two largest ethnic groups are of White British and Pakistani origin. At the time this study was conducted, only three studies on feeding practices between Pakistani and White British mothers in the UK were found and their findings were somewhat conflicting. Bowes and Domoko [29] found that White British mothers were more likely to negotiate and maintain breastfeeding than Pakistani mothers, whereas Kelly and Lee [30,31] found that South Asian and Black mothers were more likely to continue breastfeeding at 3 months compared with White mothers. None of these studies looked at factors affecting intention to mixed-feeding, particularly during the early feeding stage. Midwives working within the Bradford local maternity unit have shared their concern that South Asian mothers insist on bottle-feeding their babies in the absence of medical indication and despite initiation of breastfeeding.

It is important to understand variations in infant feeding by ethnicity as research on this topic might imply differing interventions for different groups. However, as ethnicity cannot be changed, it is also important to understand the modifiable determinants of breastfeeding behaviour. Modifiable behaviour factors have been widely explored using the Theory of Planned Behaviour (TPB) [32] that states that behaviour is determined by intention (motivation) and perceived behavioural control (PBC, the extent to which an individual feels able to perform a behaviour – closely linked to Bandura’s concept of self-efficacy [33]). Intention, in turn is predicted by attitudes (the extent to which a behaviour is evaluated as positive or negative), and subjective norms (the extent to which others would approve of the behaviour-injunctive norms, or do the behaviour themselves – descriptive norms). The TPB has good explanatory power, typically explaining around 19% variance in prospective behaviour, and 44% variance of intention for general health behaviour [34].

A number of studies have explored the ability of the TPB to predict breast and bottle-feeding [18,19,30,35] and some have controlled for the influence of deprivation or ethnicity. In the UK for example, McMillan et al. [36,37] found education to be predictive of breastfeeding intentions in addition to TPB variables, but not predictive of bottle-feeding intentions. Lawton et al. [38] found that ethnicity was predictive of intention to breastfeed in addition to TPB variables (perceived behavioural control, attitudes and descriptive norms), but that only education, affective attitude and intention were predictive of subsequent initiation. To our knowledge no paper has explicitly explored the different intentions to feed together, and their predictive factors, within a bi-ethnic deprived population. This study used a multi-methods approach [39] and had the following two specific objectives: (1) To identify which demographic, socioeconomic and TPB-related factors are associated with differences in feeding intentions; and (2) To identify reasons underpinning different feeding intentions (breastfeed, bottle-feed and mix-feed) as seen through women’s own understandings and experiences of this matter.

## Methods

### Study design

This study was nested within the Born in Bradford cohort study. Born in Bradford (BiB) is a longitudinal multi-ethnic birth cohort study aiming to examine the impact of multiple factors on maternal and child health and wellbeing [40]. The cohort is broadly characteristic of the city’s maternal population. Bradford is a city in the North of England with high levels of socioeconomic deprivation and ethnic diversity.

Women were recruited and completed a baseline questionnaire including detailed information on socioeconomic characteristics and ethnicity at approximately 28 weeks gestation whilst attending a routine clinical appointment at the city’s main maternity unit. Full details of sampling and recruitment strategies can be found in [40]. Ethical approval for the data collection was granted by Bradford Research Ethics Committee (Ref 07/H1302/112).

### Sample

The sample consisted of 555 pregnant women who were invited to participate in this nested study between October and December 2010. These women were asked to complete an additional questionnaire assessing theory of planned behaviour (TPB) variables in relation to their choice of feeding intention. A total of 532 completed the questionnaire (it was not possible to recruit 23 women who did not have a good standard of spoken English) and 476 had complete information on all variables of interest and were considered for analysis.

### Outcome measure

Feeding intention was derived from a question asking women to report the degree of their intention to breastfeed, bottle-feed and mixed-feed using a 5 category Likert scale ranging from “definitely not” [1] to “definitely yes” [5]. Women who reported high intention to breastfeed (4 or 5 from the Likert scale; scoring 1) and simultaneously low intention to both bottle and mixed-feed (1,2 or 3 from the Likert scale; scoring 0) were labelled as *“high intention to breastfeed”.* The same process was repeated for bottle-feeding (*“high intention to bottle-feed”)* and mixed-feeding *(“high intention to mixed-feed”)*. Women without any clear high intention to any feeding practice or scoring high in more than one feeding practice were labelled as *“no clear intention”*.

### Demographic characteristics

Five variables relevant to breastfeeding in previous research were considered for analysis [7]. These variables were:

a) Ethnicity (self-assigned using the 2001 UK census classification [41] and categorised into White British, Pakistani, and Other);

b) Age at pregnancy booking (continuous variable);

c) Body Mass Index at booking (BMI; extracted from the hospital’s maternity IT system and categorised according to the World Health Organization definitions [42] into <25, 25–29.9, ≥30);

d) Marital and cohabitation status (married/living with partner and single/not living with partner); and

e) Parity (binary variable: primiparous or multiparous)

### Socioeconomic factors

These were:

a) Mothers educational level. Educational status was chosen as a proxy measure of socioeconomic status (SES). Other possible measures of SES like the Index of Multiple Deprivation (IMD) or the National Statistics Socioeconomic Classification (NS-SEC) are based on occupation and thus not applicable to large proportion of our sample of women as they do not work. In England pupils sit General Certificate of Secondary Education (GCSE) examinations in different subjects usually at age 14–16, receiving 5 or more GCSEs is usually a requirement for undertaking Advanced level (A-level) studies, which are examinations in different subjects usually taken at age 16–18 before attending university. We equivalised the mother’s highest educational qualifications (based on the qualification received and the country obtained) into one of five categories using UK National Academic Recognition Information Center [43]: less than 5 GCSE equivalent, 5 GCSE equivalent, A level equivalent, Higher than A level, other);

b) Measures of financial security [44]. These were used as proxy indicators of deprivation as traditional indexes of deprivation (e.g. the Index of multiple deprivation [45]) show very little variation in the current deprived sample (e.g. where 85% of women were classed in the bottom two quintiles) These measures were:

a. Ability to pay the bills (yes/no);

a. Doing financially worse-off than a year ago (yes/no);

a. Receiving means-tested benefit (yes/no).

### Theory of planned behaviour variables (TPB)

TPB variables were assessed in relation to intended method of feeding baby in the first 2–3 weeks, as this type of feeding practice used early after delivery, particularly breastfeeding, has proven to be a key indicator for future feeding practices and child’s health and development [4]. Unless otherwise indicated all questions were answered on five point likert type scales with higher scores indicating more positive attitudes, greater social norm and greater self-efficacy.

1) Attitudes: three items assessed women’s global attitude toward ‘breastfeeding’, ‘mixed-feeding (breast and bottle)’, and ‘bottle/formula-feeding’ in the first 2–3 weeks after birth (e.g. my attitude towards breastfeeding in the first 2–3 weeks after birth is (1) very negative to (5) very positive).

2) Injunctive social norms: three items assessed whether women felt pressure to breastfeed, mixed-feed or bottle/formula feed in the first 2–3 weeks (e.g. I feel under pressure to [breastfeed only in the first 2–3 weeks] by family and friends (1) definitely not to (5) definitely yes).

3) Descriptive social norms: one question assessed ‘how other mothers you know (friends or relatives) fed their babies in the first 2–3 weeks’. Participants could select one of four options: ‘most of them breastfeed only’, ‘most of them bottle-feed only’, ‘most of them mixed-feed (breast and bottle)’, and ‘don’t know/not had much contact with new parents’.

4) Self-efficacy: three items assessed ‘how easy or difficult’ it would be for them to breast, bottle and mixed-feed their babies in the first 2–3 weeks (e.g. how easy to do you think it would be to breastfeed your baby/babies in the first 2–3 weeks: (1) very difficult to (5) very easy).

### Statistical analysis

#### Quantitative analysis

Descriptive statistics for socio-demographic and TPB factors are reported as means for continuous variables and proportions for categorical variables (Table 1). We conducted unadjusted multinomial regression models to explore the relationship between type of feeding intention and each demographic, socioeconomic and TPB factor individually; for parsimony these results are presented, but not interpreted in the results section (Table 2). To avoid spurious associations due to multiple-comparison testing [46], a final model on the association between intention to feed and all TPB measures altogether was adjusted only for potential confounders that showed a significant association with intention (ethnicity, education, and receiving means-tested benefits). Age, parity, marital and co-habitation status, ability to pay the bills and current financial situation were excluded from the final adjusted model as they were not significantly associated to intention in this fully adjusted model (Table 3). This adjusted model was estimated through manual stepwise analysis, carefully allowing for each covariate to be included and excluded from the model. The proportion of variance explained of the model was estimated using the pseudo-R2. Given the cross-sectional nature of this study, we present prevalence rate ratios (PRR) instead of relative risk ratios, with 95% confidence intervals (95% CI). Two tailed P values of less than 0.05 were considered to be significant. Details of other analysis are available from the first author on request. Analyses were conducted in Stata Software version 12 [47].

**Table 1:** Characteristics of the study sample, overall and by type of feeding intended to practice during the first 2–3 weeks after birth

	**Total sample (n = 476)**	**Type of feeding intended to practice during the first 2–3 weeks after birth**			
		**High intention to breastfeed (n = 258)**	**High intention to mixed-feed (n = 50)**	**High intention to bottle feed (n = 80)**	**No high intention for any feeding practice (n = 88)**
***Demographic and Socioeconomic factors***					
Ethnicity:					
White British	219 (46.0)	105 (40.7)	19 (38.0)	63 (78.5)	32 (36.3)
Pakistani	157 (32.9)	83 (32.1)	23 (46.0)	13 (16.2)	38 (43.1)
Other	100 (21.0)	70 (27.1)	8 (16.0)	4 (5.0)	18 (20.4)
Mean (SD) age (years)	28.0 (5.6)	28.5 (5.4)	28.4 (5.4)	26.4 (5.5)	27.8 (6.2)
Parity:					
Primiparous	257 (54.1)	149 (58.0)	27 (54.3)	32 (40.0)	47 (54.4)
Multiparous	219 (45.9)	108 (41.9)	23 (45.6)	48 (60.0)	41 (45.5)
BMI:					
Normal (BMI <25)	130 (27.8)	67 (25.9)	13 (26.0)	28 (35.0)	22 (25.0)
Overweight (BMI 25–29.9)	176 (36.9)	103 (39.6)	23 (46.0)	19 (23.7)	31 (35.2)
Obese (BMI ≥30)	170 (35.7)	88 (34.11)	14 (28.0)	33 (41.2)	35 (39.7)
Marital and cohabitation status:					
Married/living with partner	379 (81.39)	217 (84.2)	45 (90.0)	48 (60.0)	69 (78.5)
Single/not living with partner	99 (18.6)	41 (15.8)	5 (10.0)	32 (40.0)	19 (21.5)
Mother’s educational level^a^:					
Less than 5 GCSE equivalent	78 (16.3)	34 (13.1)	9 (18.0)	18 (22.5)	17 (19.3)
5 GCSE equivalent	132 (27.3)	55 (21.3)	13 (26.0)	37 (46.2)	27 (30.6)
A level equivalent	90 (18.9)	45 (17.4)	14 (28.0)	14 (17.5)	17 (19.3)
Higher than A level equivalent	145 (18.9)	107 (41.4)	14 (28.0)	4 (5.0)	20 (22.7)
Can’t pay the bills	53 (11.1)	29 (9.9)	7 (12.8)	10 (11.7)	14 (14.9)
Finances worse than a year ago	115 (24.1)	68 (26.3)	10 (20.0)	18 (22.5)	19 (21.5)
Receiving means-tested benefit	181 (38.0)	86 (33.3)	11 (22.0)	50 (62.5)	34 (38.6)
***Theory of planned behaviour factors***					
Mean (SD) attitude scores^1^					
To breastfeed only	4.1 (1.0)	4.7 (0.7)	3.8 (0.9)	2.5 (0.8)	4.0 (0.9)
To Mixed-feed	3.1 (1.0)	3.0 (0.9)	3.9 (0.8)	2.5 (0.9)	3.6 (0.9)
To bottle-feed only	3.0 (1.2)	2.4 (1.0)	2.8 (0.9)	4.5 (0.5)	3.2 (0.9)
Mean (SD) injunctive norm to breastfeed^2^	1.7 (1.9)	1.7 (1.2)	1.7 (1.2)	1.6 (0.9)	1.9 (1.3)
Mean (SD) injunctive norm to mixed-feed^2^	1.6 (1.0)	1.5 (0.9)	1.7 (0.9)	1.6 (1.0)	1.8 (1.0)
Mean (SD) injunctive norm to bottle-feed^2^	1.4 (0.8)	1.3 (0.8)	1.5 (0.9)	1.3 (0.7)	1.4 (0.7)
Descriptive norms (categorical)^3^					
Other people breastfeed only	143 (30.0)	106 (41.0)	5 (10.0)	2 (2.5)	30 (34.0)
Other people mixed-feed	151 (31.7)	76 (29.4)	29 (58.0)	19 (23.7)	27 (30.6)
Other people bottle-feed only	156 (32.7)	66 (25.5)	13 (26.0)	51 (63.9)	26 (29.5)
Mean (SD) Self-efficacy scores^1^					
To breastfeed only	3.1 (1.2)	3.5 (1.0)	2.9 (1.0)	1.8 (0.9)	3.0 (1.1)
To mixed-feed	2.5 (1.4)	2.4 (1.4)	3.3 (1.4)	1.8 (1.1)	2.9 (1.3)
To bottle-feed only	3.2 (1.7)	2.9 (1.7)	2.9 (1.9)	4.4 (0.8)	3.5 (1.5)
Had previous negative experience of breastfeeding:					
No previous experience	256 (54.0)	276 (58.0)	139 (54.0)	32 (40.0)	48 (54.0)
Negative experience	130 (26.0)	119 (23.0)	39 (15.0)	37 (47.0)11 (12.0)	24 (26.0)
Positive experience	90 (19.0)	81 (18.0)	80 (30.0)		16 (19.0)

^a^GCSE = General Certificate of Secondary Education; A-level = Advanced level.

^1^A higher score indicates more positive/higher attitude/self-efficacy with a particular feeding behaviour.

^2^Injunctive norms refer to what other people think you should do; a higher score indicates higher pressure towards certain behaviour.

^3^Descriptive norms refer to what other people actually do.

Values are frequency (percentage) unless otherwise indicated.

**Table 2:** Unadjusted multinomial regression analyses for factors potentially associated with different feeding intentions

	**High intention to mixed-feed (n = 50)**	**High intention to bottle feed (n = 80)**	**No high intention for any feeding practice (n = 88)**
Ethnicity:			
White British	1.00	1.00	1.00
Pakistani	1.41 (0.75-2.66)	0.25 (0.14-0.46)*	1.31 (0.78-2.21)
Other	0.73 (0.26-1.52)	0.09 (0.03-0.27)**	0.89 (0.46-1.69)
Mean (SD) age (years)	0.98 (0.93-1.03)	0.93 (0.89-0.98)*	0.98 (0.94-1.04)
Parity (multiparous compared to primiparous)	1.16 (0.66-2.06)	2.09 (1.23-3.42)**	1.18 (0.74-1.87)
BMI:			
Normal (BMI <25)	1.00	1.00	1.00
Overweight (BMI 25–29.9)	1.06 (0.52-2.14)	0.53 (0.28-1.00)*	1.03 (0.58-1.86)
Obese (BMI ≥30)	0.96 (0.46-2.13)	1.03 (0.58-1.25)	1.30 (0.72-2.33)
Marital and cohabitation status:	0.55 (0.21-1.47)	3.49 (2.02-6.03)**	1.39 (0.76-2.52)
Married/living with partner	1.00	1.00	1.00
Single/not living with partner	0.55 (0.21-1.47)	3.49 (2.02-6.03)**	1.39 (0.76-2.52)
Mother’s educational level^a^:			
Less than 5 GCSE equivalent	2.40 (1.07-5.35)*	8.96 (3.17-15.75)**	2.68 (1.36-5.31)**
5 GCSE equivalent	1.69 (0.76-3.78)	13.24 (14.96-25.31)**	2.54 (1.33-4.84)**
A level equivalent	2.43 (1.07-5.51)*	6.82 (2.31-20.05)**	2.06 (0.99-4.30)
Higher than A level equivalent	1.00	1.00	1.00
Can’t pay the bills	1.26 (0.52-3.06)	1.20 (0.56-2.59)	1.51 (0.76-2.99)
Finances worse than a year ago	0.78 (0.39-1.56)	0.84 (0.47-1.50)	0.80 (0.46-1.39)
Receiving means-tested benefit	0.67 (0.35-1.27)	3.13 (1.89-5.16)**	1.25 (0.78-2.02)
Mean (SD) attitude scores^1^			
To breastfeed only	0.20 (0.13-0.31)**	0.04 (0.02-0.08)**	0.27 (0.19-0.48)**
To mixed-feed	3.02 (2.03-4.49)**	0.59 (0.46-0.77)**	2.10 (1.51-2.92)**
To bottle-feed only	1.46 (1.07-1.98)*	13.57 (8.41-21.90)**	2.33 (1.74-3.12)**
Mean (SD) injunctive norm to breastfeed^2^	0.97 (0.74-0.87)	0.87 (0.70-1.09)	1.12 (0.93-1.36)
Mean (SD) injunctive norm to mixed-feed^2^	1.12 (0.84-1.49)	1.09 (0.85-1.40)	1.24 (0.98-1.56)
Mean (SD) injunctive norm to bottle-feed^2^	1.22 (0.87-1.72)	0.86 (0.44-1.25)	1.13 (0.87-1.48)
Descriptive norms score^3^			
Other people breastfeed only	1.00	1.00	1.00
Other people mixed-feed	4.57 (0.22-9.17)	5.57 (1.48-11.00)**	1.04 (0.48-2.23)
Other people bottle-feed only	7.72 (0.40-14.13)	3.08 (0.28-13.00)	1.41 (0.39-5.09)
Mean (SD) self-efficacy scores^1^			
To breastfeed only	0.58 (0.43-0.77)**	0.20 (0.14-0.29)**	0.64 (0.51-0.81)**
To Mixed-feed	1.71 (1.26-2.32)**	0.72 (0.62-0.85)**	1.33 (1.10-1.61)**
To bottle-feed only	**1.03 (0.83-1.22)**	2.36 (1.71-3.24)**	1.22 (1.05-1.41)*
Previous negative experience of breastfeeding:			
No previous experience	1.00	1.00	1.00
Negative experience	0.73 (0.33-1.63)	2.98 (1.74-5.11)**	1.24 (0.71-2.15)
Positive experience	1.69 (0.87-3.29)	1.0 (0.47-2.10)	1.10 (0.60-2.03)

^a^GCSE = General Certificate of Secondary Education; A-level = Advanced level.

^1^A higher score indicates more positive/higher attitude/self-efficacy with a particular feeding behaviour.

^2^Injunctive norms refer to what other people think you should do; a higher score indicates higher pressure towards certain behaviour.

^3^Descriptive norms refer to what other people actually do.

*p-value < 0.05, **p-value < <0.001.

High intention to breastfeed is the reference category). Values are prevalence rate ratios (PRRs) with 95% robust confidence intervals.

**Table 3:** Adjusted multinomial regression analyses for factors associated with different feeding intentions

	**High intention to mixed-feed (n = 50)**	**High intention to bottle feed (n = 80)**	**No high intention for any feeding practice (n = 88)**
Ethnicity:			
White British	1.00	1.00	1.00
Pakistani	5.36 (1.59-17.99)**	0.01 (0.001-0.12)**	2.39 (1.02-5.59)*
Other	1.05 (0.27-4.05)	0.03 (0.003-1.32)	1.84 (0.72-4.69)
Mother’s educational level^a^:			
Less than 5 GCSE equivalent	2.86 (0.81-10.07)	0.20 (0.10-3.30)	1.90 (0.56-6.42)
5 GCSE equivalent	1.57 (0.44-5.52)	12.54 (0.30-28.35)	2.90 (1.18-7.13)**
A level equivalent	2.03 (0.52-7.98)	23.40 (1.48-36.75)*	1.65 (0.61-4.45)
Higher than A level equivalent	1.00	1.00	1.00
Receiving means-tested benefit	0.22 (0.05-0.90)*	7.75 (1.55-38.58)**	0.78 (0.35-1.76)
Mean (SD) attitude scores^1^			
To breastfeed only	0.16 (0.08-0.36)**	0.04 (0.005-0.18)**	0.27 (0.16-0.45)**
To mixed-feed	3.53 (1.95-6.40)**	0.19 (0.06-0.61)**	2.02 (1.23-3.31)**
To bottle-feed only	1.05 (0.65-1.67)	52.70 (12.44-92.22)**	1.86 (1.25-2.77)**
Mean (SD) injunctive norm to breastfeed^2^	0.69 (0.31-1.54)	1.30 (0.60-2.74)	0.82 (0.48-1.17)
Mean (SD) injunctive norm to mixed-feed^2^	0.90 (0.33-2.41)	13.05 (1.99-45.45)**	1.46 (0.88-2.42)
Mean (SD) injunctive norm to bottle-feed^2^	1.46 (0.77-2.77)	0.29 (0.05-1.51)	0.95 (0.60-1.49)
Descriptive norms score^3^			
Other people breastfeed only	1.00	1.00	1.00
Other people mixed-feed	13.77 (3.38-45.57)**	0.01 (0.001-1.04)	0.64 (0.26-1.24)
Other people bottle-feed only	10.68 (2.19-32.05)**	0.16 (0.07-3.48)	0.91 (0.38-2.18)
Mean (SD) self-efficacy scores^1^			
To breastfeed only	0.62 (0.37-1.07)	0.10 (0.02-0.50)**	0.73 (0.50-1.06)
To mixed-feed	1.80 (1.07-3.04)*	5.50 (1.41-12.53)**	1.27 (0.91-1.77)
To bottle-feed only	0.70 (0.43-1.14)	0.68 (0.68-1.65)	0.97 (0.71-1.34)
Previous negative experience with breastfeeding:			
No previous experience	1.00	1.00	1.00
Negative experience	1.40 (0.35-5.62)	23.44 (2.44-55.20)**	2.09 (0.84-5.22)
Positive experience	4.87 (1.42-16.67)**	0.04 (0.01-1.12)	1.91 (0.75-4.86)

^a^GCSE = General Certificate of Secondary Education; A-level = Advanced level.

^1^A higher score indicates more positive/higher attitude/self-efficacy with a particular feeding behaviour.

^2^Injunctive norms refer to what other people think you should do; a higher score indicates higher pressure towards certain behaviour.

^3^Descriptive norms refer to what other people actually do.

*p-value < 0.05, **p-value < <0.001.

^α^Age, marital and co-habitation status, ability to pay the bills and current financial situation were excluded from the final adjusted model as they were not significantly associated to intention in this fully adjusted model. Parity was excluded from this model due to high collinearity with previous breastfeeding experience.

High intention to breastfeed is the reference category. Values are prevalence rate ratios (PRRs) with 95% robust confidence intervals^α^.

#### Qualitative analysis

Qualitative data were analysed using thematic content analysis [48] according to each feeding intention group. The ‘no clear intention’ group was excluded from the qualitative analysis due to the difficulties inherent in assigning reasons behind intention where no clear intention was specified. One coder (DW) coded each reason (N = 871) according to underlying meaning (e.g. ‘for babies immunity’, ‘good immunity’ were coded as ‘good for babies immunity’). A random 10% was checked by a second coder (RM) and agreement was 100%. Both coders then independently grouped the codes into themes, agreed a final list of themes, and then independently recoded each reason into one of the agreed themes, 35 reasons were coded into two themes. Initial agreement was 85.3% (N = 743). After discussion and clarification, further 63 reasons were coded into agreed themes, and a new theme created for a further 51 reasons. Agreements could not be reached for 14 reasons and were arbitrated by a third independent coder (BC). In order to aid interpretation, themes were grouped into higher order ‘categories’ that were linked to each type of intended feeding practice.

## Results

### Quantitative results

Participants reported a mean age of 28 (SD 5.6), about 72% were either overweight (BMI between 25–30) or obese (BMI > 30) at booking, and the majority were married (60%) and lived with a partner (80%). Around 54% of the sample was primiparous and 45% multiparous. Primiparous women were more likely to report breastfeeding (58%) and mixed-feeding (54%) intention, whereas multiparous women reported a higher intention to bottle-feed (60%). Most women reported a 5 GCSE equivalent education level (27%), followed by A level and higher than A level (18% each, respectively), and less than 5 GCSE equivalent (16%). Just under a 40% of the sample reported receiving a means-tested benefit, 24% stated being financially worse than a year ago and 11% reported not being able to pay the bills. Forty-six percent of the sample were White British, 32% were Pakistani and 21% were of ‘Other’ ethnic origin.

The majority of the sample (54%) were classified as ‘breastfeeding intenders’, 17% were categorised as ‘bottle-feeding intenders’ and 10% were categorised as ‘mixed-feeding intenders’, see Table 1. Around 20% of the sample reported no clear intentions. Breastfeeding intenders reported more positive attitudes towards breastfeeding (mean 4.7 (SD 0.7)), than mixed-feeding intenders (3.8 (0.9)) or bottle-feeding intenders (2.5 (0.8)). Mixed-feeding intenders showed more positive attitudes towards mixed-feeding (3.9 (0.8)) than breastfeeding and bottle-feeding intenders; and the same patterns were observed for bottle-feeding intenders. Positive injunctive norms were not clearly patterned. However, self-efficacy scores and descriptive norms were higher for each intended feeding practice. Around 54% of the sample had no previous negative experience with breastfeeding, 26% reported having had a negative experience and 19% reported a positive past experience.

Table 2 shows the unadjusted models and Table 3 shows the final model explaining the association between feeding intentions and all TPB factors integrated in a single model (adjusted by age and ethnicity). The final model explained 54% variance in feeding intention, and showed an adequate statistical fit. Generally, TPB variables were significantly associated with feeding intentions in pregnant women, even after controlling for ethnic background and socioeconomic factors. Mixed-feeding intenders were more likely to be Pakistani (PRR: 5.36, 95% CI: 1.59-17.99), to have negative attitudes towards breastfeeding (PRR: 0.16, 95% CI: 0.08-0.36) and positive attitudes towards mixed-feeding (PRR: 3.53, 95% CI: 1.95-6.40). They reported more often that other people tend to mixed-feed (PRR: 13.77, 95% CI: 3.38-45.57) and bottle-feed (PRR: 10.68, 95% CI: 2.19-32.05) compared to the’breastfeeding intender group’. They were also more likely to report previous positive experience with breastfeeding (PRR: 4.87, 95% CI: 1.42-16.67).

The bottle-feeding group were less likely to be Pakistani (PRR: 0.01, 95% CI: 0.001-0.12), and were more likely to have lower levels of education (A-level equivalent compared with higher than A level equivalent, PRR: 23.40, 95% CI: 1.48-36.75). This group reported more negative attitudes towards breastfeeding (PRR: 0.04, 95% CI: 0.005-0.18) and mixed-feeding (PRR: 0.19, 95% CI: 0.06-0.61), and more positive attitudes towards bottle-feeding (PRR: 52.70, 95% CI: 12.44-92.22). They also reported lower self-efficacy towards breastfeeding (PRR: 0.10, 95% CI: 0.02-0.50), and higher self-efficacy and injunctive norms toward mixed-feeding (PRR: 5.55, 95% CI: 1.41-12.53 and PRR 13.05, 95% CI: 1.99-45.45 respectively). Bottle-feeding intenders were also more likely to report negative previous experiences with breastfeeding (PRR: 23.44, 95% CI: 2.44-55.20).

Finally, women with no clear intention were more likely to be Pakistani (PRR: 2.39, 95% CI: 1.02-5.59), to have GSCE level education compared with higher than A level education (PRR: 2.69, 95% CI: 1.18-7.13) and to report positive attitudes towards mixed-feeding (PRR: 2.02, 95% CI: 1.23-3.31) and bottle-feeding (PRR: 1.86, 95% CI: 1.25-2.77). They were less likely to report positive attitudes towards breastfeeding (PRR: 0.27, 95% CI: 0.16-0.45).

### Qualitative results

Overall 871 reasons for feeding intention were provided. 244 women in the breastfeeding intention group provided a total of 648 reasons for their choice (average 2.7 per woman, 35 of these could be coded in two separate themes giving 683 codes for this group); 41 women in the mixed-feeding intention group provided on average 2.5 reasons (total 102 reasons); and 59 women in the bottle-feeding group provided on average 2 reasons (total 121 reasons). A summary of the content analysis can be found in Table 4.

**Table 4:** Summary table of content analysis of reasons behind feeding intentions of study participants

**Type of feeding**	**Category**	**Themes (in descending order of frequency)**	**Number of codes**	**Percentage of intention category**
***BREASTFEEDING***				
Breastfeeding reasons (N = 244 women, n = 648 reasons)*	Breast is best	• Healthy/good for baby and/or mum	403	59.0%
		• Nutritious food for baby		
*NB 35 reasons were coded in 2 categories		• Help mum to lose weight		
	Emotions	• Helps bonding	132	19.3%
		• Natural		
		• Comfortable		
		• Happy baby/mother		
		• Negative feelings about formula		
		• Happy		
	Easier	• Convenient	103	15.1%
		• Easier		
		• Cheaper		
		• Hygienic		
	Social norms	• Influence of others	12	1.8%
		• Moral duty		
	Past behaviour	• Breast fed previous child	8	1.2%
	Motivation	• I want to breastfeed	6	0.9%
	Uncertainty	• May not work	4	0.6%
	Could not code		15	2.2%
	***TOTAL BREASTFEEDING***		***683***	***100%***
***MIXED-FEEDING***				
	***Breastfeeding reasons within Mixed-feeding intentions***			
Mixed-feeding reasons: N = 41 women, n = 102 reasons	Breast is best	• Healthy/good for baby and/or mum	47	46.1%
		• Nutritious food for baby		
		• Help mum to lose weight		
	Emotions	• Helps bonding	19	18.6%
		• Natural		
	Easier	• Cheaper	5	4.9%
		• Convenient		
		• Easier		
	Social norms	• Influence of other people	2	2.0%
	Motivation	• I want to give breast milk	1	1.0%
	***Total breastfeeding related reasons***		**74**	**72.50%**
	***Bottle-feeding reasons within Mixed-feeding intentions***			
	Confidence	• Knowing that baby is full	10	9.8%
		• Uncertainty about ability to breastfeed		
	Easier	• Allows others to feed baby	4	3.9%
		• Convenient		
		• Easier		
	Emotions	• Breastfeeding affects appearance of breast	3	2.9%
		• Allows others to bond with baby		
	***Total bottle-feeding related reasons***		**17**	**16.7%**
	Could not code		11	10.2%
	***TOTAL MIXED-FEEDING***		***102***	***100%***
***BOTTLE-FEEDING***				
Bottle-feeding reasons, N = 59 women, n = 121 reasons)	Easier	• Allow others to feed baby	39	32.2%
		• Easier		
		• Convenient		
		• Routine		
	Emotions	• Negative experience of breastfeeding	27	22.3%
		• Allow others to bond		
		• Comfortable		
		• Other child doesn’t feel excluded		
	Confidence	• Knowing baby is full	20	16.5%
		• Confidence in bottle-feeding		
		• Uncertainty about ability to breastfeed		
	Motivation	• I don’t want to breastfeed	13	10.7%
		• I want to bottle-feed		
	Past behaviour	• Bottle fed previous child	7	5.8%
	Good	• Bottle-feeding good for baby	2	1.7%
	Social norms	• Influence of other people	2	1.7%
	Uncertainty	• May change my mind	2	1.7%
	Control	• Might have no control over breastfeeding (e.g. unable for medical reasons)	1	0.8%
	Could not code		8	6.6%
	***TOTAL BOTTLE-FEEDING***		***121***	***100%***
	***GRAND TOTAL***		***871***	

*35 reasons were coded in 2 categories.

For the high breastfeeding intenders group we found 18 themes, which were grouped into 7 broader categories. The most common categories were: (i) breastfeeding is the best (59.0% of reasons, e.g. “*it is healthy for the baby and the mum*”), (ii) it is closely related to positive emotions (19.3%, e.g. “*helps bonding*”, “*it makes me happy*”), (iii) it is easier (15.1%, e.g. “*it is cheaper*”, “*it is more hygienic*”). Thus the majority of reasons for the choice of breastfeeding appeared to capture tangible (instrumental) benefits of the behaviour such as health benefits and convenience (64% of all reasons). In addition, 20% of reasons focused around emotional reasons for breastfeeding (e.g. bonding).

The mix-feeding intenders were a complex group, as these women reported reasons for breast (72.5% of reasons) and bottle-feeding (16.7% of reasons) simultaneously, implying that women create an intention to mixed-feed from an analysis of the benefits of both bottle and breastfeeding, rather than thinking of mixed-feeding as a distinct behaviour. Themes and categories identified for breast and bottle-feeding were similar to the other groups.

Reasons amongst the bottle-feeding intenders group were more disparate and were organised into 18 themes, within 9 categories. The most common of these were: (i) it is easier (32.2% of reasons, e.g. *“easier to share feeding with partner”,* “*it is more convenient*”, “*it is part of my routine*”), ii) relations with both negative emotions (22.3%, e.g. negative experience with breastfeeding *“found it difficult and distressing last time”),* and positive emotions associated with involving others in feeding (e.g. *“helps the father bond with the baby”, “my other children don’t feel excluded”),* iii) confidence relating to bottle-feeding compared with breastfeeding (16.5%, e.g. *“wasn’t sure how much the baby was drinking, this allow me to know for sure”,* “*I am confident I can bottle-feed my baby*”), iv) linked to women’s motivations (10.7%, e.g. “*I don’t want to breastfeed*”, “*I just want to Bottle-feed”*), v) past behaviour (5.8%, e.g. *“that’s what I did with my other two”).* Similar proportions of affective reasons (e.g. emotions and confidence, 38%), compared with more pragmatic, instrumental consideration (e.g. convenience, 32%) were highlighted for this group.

## Discussion

Our analyses showed that generally feeding intention was significantly predicted by modifiable variables from the theory of planned behaviour (TPB) such as attitudes, descriptive norms and self-efficacy, controlling for key demographic or socioeconomic factors. Compared to breastfeeding intenders, Pakistani women were more likely to intend to mixed-feed (PRR = 5.36) or have no clear intention (PRR = 2.39).

Concerning TPB factors, generally attitudes were consistently predictive for each intention group. Compared with breastfeeding intenders, mixed-feeding intenders reported more negative attitudes towards breastfeeding (PRR = 0.16), and more positive attitudes towards mixed-feeding (PRR = 3.53). Bottle-feeding intenders reported negative attitudes towards breastfeeding and mixed-feeding (PRR = 0.04 and 0.19 respectively), and more positive attitudes towards bottle-feeding (PRR = 52.70). Self-efficacy was an important predictor of mixed and bottle-feeding intention. Interestingly, bottle-feeding intention was not predicted by self-efficacy in relation to bottle-feeding, but by lower self-efficacy for breastfeeding only (PRR = 0.10), and higher self-efficacy for mixed-feeding (PRR = 5.50).

A similar pattern was apparent for mixed-feeding intention, although the tendency to report lower self-efficacy for breastfeeding was not significant. A role for subjective norms was also apparent. Descriptive norms for mixed (PRR = 13.77) and bottle-feeding (PRR = 10.68) were predictive of mixed-feeding intention, and injunctive norms towards mixed-feeding were predictive of bottle-feeding intention (PRR = 13.05). Unsurprisingly, past experience with breastfeeding had an important role to play. Women who reported a negative past experience with breastfeeding (compared with those with no prior experience) were more likely to intend to bottle-feed.

The qualitative analysis helped to add depth to these findings. Reasons for breastfeeding intentions generally related to health considerations, whilst bottle-feeding reasons related to convenience, and in certain cases alluded to negative past experiences (e.g. pain when Breastfeeding) or issues surrounding confidence in breastfeeding (e.g. not knowing whether baby would get enough milk).

Our findings are generally consistent with other studies in this area. Lawton et al. [38] found roles of attitudes and self-efficacy in the prediction of breastfeeding intention, although this study did not assess other feeding intentions. McMillan et al. [36] found that positive breastfeeding attitude, norms and control were predictive of breastfeeding intentions, and that positive bottle-feeding attitude and perceived behavioural control were predictive of formula feeding intentions. However, this study did not explore how beliefs towards different behaviours (in this case bottle-feeding and breastfeeding) impact in tandem to create feeding intentions. Thus a major contribution of this study is to highlight the complex evaluation of the pros and cons of different feeding options that occurs when women make a feeding intention during pregnancy. It is important that clinicians understand the factors underlying motivation to engage in feeding practices such as mixed or bottle-feeding in order to ascertain where best to focus efforts for intervention.

It would appear from our results that different intervention strategies are likely to be important for different groups. Whilst all groups would benefit from an increase in positive attitude towards breastfeeding, bottle-feeding intenders and those with no clear intention might benefit from more intensive attempts to build self-efficacy in relation to breastfeeding (see [49,50]). In line with clinician expectations, we did find a small group of mothers with mixed-feeding intentions (10%), who were more likely to be of Pakistani origin. Interestingly, the reasons behind their choice of mixed-feeding seemed to centre on the positive benefits of breastfeeding. The fact that descriptive norms for mixed-feeding were significantly predictive of this group (in addition to descriptive norms for bottle-feeding) might be an indication that for this cultural group, mixed-feeding is perceived as ‘normal’ practice, and that this social pressure has an impact on how women intend to feed their child [51,52]. Interventions amongst these groups should recognise the need for culturally sensitive information on feeding practices to be extended to the social network of pregnant women.

Future research should explore experiences of mixed-feeding intenders over time and the extent to which early introduction of mixed-feeding impacts on how long a woman successfully continues breastfeeding. This should be explored in line with her expectations and how to better address pregnant women’s concerns about feeding practices, ideally including their partners and relatives. Our findings also appear to suggest that we need to target fathers and encourage their involvement in parenting activities other than feeding and to help them better support breastfeeding. The opinions and beliefs of women’s close ones might be more important than initially thought for women’s final decisions on feeding practices and clinicians need to take this aspect into consideration.

The current study has a number of strengths. To our knowledge we are the first study to explicitly explore factors related to intentions to mix-feed in a large bi-ethnic sample, in addition to other feeding practices. Our multi-method approach allowed us to explore in greater detail motivations behind different feeding practices, and we were able to control for a wider range of socioeconomic variables than previous research (for example [36-38,51]). However, there are limitations. It was difficult to recruit immigrant women without a good standard of spoken English and, as a consequence, we might have missed some differences in TPB factors related to feeding intentions for this particular group. Future research should also explore factors such as acculturation (taking into account language abilities) and how they relate to ethnic differences [53-55]. TPB variables for each feeding method were assessed using self-reported single indicators instead of more complex scales meaning reliability cannot be assessed, although single item measures have proven to be useful and meaningful indicators of other complex constructs (e.g. self-esteem [56]). The questionnaire was cross-sectional, assessing feeding intentions, and not actual behaviour. The final adjusted model explained 54% variance in feeding intention, and showed an adequate statistical fit. However, due to small numbers in some categories (e.g. some categories of ethnicity, marital and cohabitation status, and educational level with less than 5 cases), some of the confidence intervals in the results section were wide. Future research with larger sample sizes within different feeding intention groups could test the robustness of these findings.

There are some important clinical implications from this study. Health professionals promote exclusive breastfeeding to pregnant women and discourage early mixed-feeding or bottle-feeding. Subsequently alternative feeding practices are rarely discussed and women are therefore not asked about their intention and motivation to mix-feed or bottle-feed, resulting in a lack of relevant information and support. The current study has highlighted in more detail the underlying motivations behind choices to mix-feed which centre around combining the perceived benefits of both behaviours (for example, health aspects of breastfeeding, with convenience aspects of bottle-feeding for example), and bottle-feed (which focus on convenience and lack of confidence in breastfeeding).

## Conclusions

The current study explored the relative prediction of ethnicity, socioeconomic status and modifiable TPB determinants in understanding mother’s intentions to breast, bottle or mixed-feed their babies in the first 2–3 weeks after birth. The results are useful in identifying beliefs to target in intervention to change women’s feeding intentions and promote breastfeeding. Clinicians aiming to encourage intentions to breastfeeding might usefully focus on the health-benefits, convenience, and bonding aspects of breastfeeding to try and encourage positive attitudes. Within our sample we found around half of women (54%) intended to breastfeed, with 17% indicating intentions to bottle-feed, and 10% indicating intentions to mixed-feed. We found that a rather large group of pregnant women (20%) were undecided about which feeding practice to choose immediately after delivery, suggesting there is a window of opportunity to promote breastfeeding among this particular group.

This study provides unique novel information about the complex process experienced by pregnant women of choosing how to feed their newborns, which should be considered to improve antenatal and postnatal counselling and practice.

## Abbreviations

TPB: Theory of planned behaviour; UK: United Kingdom; PBC: Perceived behavioural control; BiB: The Born in Bradford cohort study; PRR: Prevalence rate ratios.

## Competing interests

The authors declare that they have no competing interests.

## Authors’ contributions

BC was involved in the article’s conception, data analysis, drafting, final review and submission. DG was involved in the study’s conception, data collection, data analysis, drafting, final review and submission. GS was involved in the article’s conception, data analysis, drafting, final review and submission. RM was involved in the article’s conception, data analysis, drafting, final review and submission. All authors read and approved the final manuscript.

## Authors’ information

Baltica Cabieses, Lecturer at Universidad del Desarrollo Clínica Alemana Chile; Associate Epidemiologist at the Bradford Institute for Health Research, Bradford Teaching Hospitals NHS Foundation Trust, Bradford Royal Infirmary England. Dagmar Waiblinger, Research community coordinator at the Bradford Institute for Health Research, Bradford Teaching Hospitals NHS Foundation Trust, Bradford Royal Infirmary England. Gillian Santorelli, Statistician at the Bradford Institute for Health Research, Bradford Teaching Hospitals NHS Foundation Trust, Bradford Royal Infirmary England. Rosemary R C McEachan, Programme manager at the Bradford Institute for Health Research, Bradford Teaching Hospitals NHS Foundation Trust, Bradford Royal Infirmary England.

## Pre-publication history

The pre-publication history for this paper can be accessed here:

http://www.biomedcentral.com/1471-2393/14/50/prepub
